# Forgivingness of an Anteromedially Positioned Small Locked Plate for High Tibial Osteotomy in Case of Overcorrection and Lateral Hinge Fracture

**DOI:** 10.3390/life12081265

**Published:** 2022-08-19

**Authors:** Sabrina Böhle, Lars Bischoff, Kristin Ehrenmann, Frank Layher, Klaus Sander, Georg Matziolis, Stefan Pietsch

**Affiliations:** 1 Orthopaedic Professorship of the University Hospital Jena, Orthopaedic Department of the Waldkliniken Eisenberg, 07607 Eisenberg, Germany; 2 Department of Surgery, Oberschwabenklinik, St. Elisabethen-Klinikum, 88212 Ravensburg, Germany

**Keywords:** HTO, high tibial osteotomy, opening wedge, lateral hinge fracture, pseudarthrosis, stiffness, failure, fixation plate, biomechanical study

## Abstract

High tibial osteotomy (HTO) represents a sensible treatment option for patients with moderate unicondylar osteoarthritis of the knee and extraarticular malalignment. The possibility of a continuously variable correction setting and a surgical approach low in complications has meant that the medial opening osteotomy has prevailed over the past decades. The objective of the present study was to determine whether anteromedially positioned small plates are nevertheless forgiving under biomechanically unfavourable conditions (overcorrection and lateral hinge fracture). In this study, a simulated HTO was performed on composite tibiae with a 10-mm wedge and fixed-angle anteromedial osteosynthesis with a small implant. Force was applied axially in a neutral mechanical axis, a slight and a marked overcorrection into valgus, with and without a lateral hinge fracture in each case. At the same time, a physiological gait with a dual-peak force profile and a peak load of 2.4 kN was simulated. Interfragmentary motion and rigidity were determined. The rigidity of the osteosynthesis increased over the cycles investigated. A slight overcorrection into valgus led to the lowest interfragmentary motion, compared with pronounced valgisation and neutral alignment. A lateral hinge fracture led to a significant decrease in rigidity and increase in interfragmentary motion. However, in no case was the limit of 1 mm interfragmentary motion critical for osteotomy healing exceeded. The degree of correction of the leg axis, and the presence of a lateral hinge fracture, have an influence on rigidity and interfragmentary motion. From a mechanically neutral axis ranging up to pronounced overcorrection, the implant investigated offers sufficient stability to allow healing of the osteotomy, even if a lateral hinge fracture is present.

## 1. Introduction

Osteoarthritis of the knee represents one of the most common joint diseases in adulthood worldwide [1,2] and occurs in the medial joint compartment in around 70% of cases [3]. This is often accompanied by a varus malalignment [4], in which there is a considerably higher load on the medial compartment in the knee joint [5]. Even with normal leg alignment, there is not a strictly half load distribution on the two joint compartments of the tibiofemoral joint during walking, but rather a predominantly medial load [6,7,8]. After the exhaustion of conservative therapeutic measures, an established joint-preserving treatment option is a medial open wedge high tibial osteotomy (HTO) for medial osteoarthritis caused by varus malalignments of the knee [9,10,11,12], which can be used for symptomatic therapy as well as to prevent secondary osteoarthritis. The aim of the high valgising medial tibial osteotomy using an open-wedge technique is to alter precisely this mechanical axis in the sense of a transfer from the degenerative medial compartment side to the unaffected lateral component [11,13]. In the opening valgus procedure, an osteotomy is placed on the medial surface of the tibia approximately 25 mm below the tibiofemoral articular surface and carefully, with the insertion of increasing wedges, the osteotomy gap is widened to the desired correction angle [14]. The main complications, beside overcorrection [15] and fractures of the lateral contralateral cortical bone (hinge fracture) [16], include non-union of the osteotomy, as well as often a mechanical contact to the pes anserinus and the medial collateral ligament [17]. In order to prevent non-union of the osteotomy gap as well as loss of correction, the extent of interfragmentary motion and the resulting rigidity of the HTO must be optimised. According to animal experiments conducted by Claes et al., an interfragmentary motion of up to 0.5 mm is ideal for the formation of a callus with maximum mechanical rigidity [18]. Since interfragmentary movements occur upon loading of the leg, despite the use of different fixation devices, investigations were devised with the aid of artificial bone (third-generation Sawbones) to measure the interfragmentary motion and the rigidity of HTO [19]. Here, when axial force was applied to the Fujisawa point at 62% [14,20], a long rigid plate (TomoFix R plate (Synthes, Oberdorf, Switzerland)) showed the lowest interfragmentary motion and the greatest resilience towards a single loading, as well as towards cyclic loading [19]. Further implants were tested, and it was found that the thickness, shape, and rigidity of the plates play a major role in bone healing after HTO [21,22,23,24,25,26].

The Activmotion^®^ osteotomy plate used here (Newclip^®^, Haute Goulaine, France) has a length 61,6 mm smaller compared to other implants, such as a TomoFix R plate with 115 mm, and is characterized by an anteromedial positioning to spare the pes anserinus [22] instead of strictly medial. In addition, proximal fixation with a polyaxial screw allows for customization of screw location into the subchondral bone. It is unclear to what extent small size and atypical implant positioning in frontal plane have an influence on interfragmentary motion, rigidity, and forgivingness towards a lateral hinge fracture. This was to be elucidated in the present study.

## 2. Materials and Methods

Five fourth-generation composite tibiae from the company Sawbones (# ERP 3401, Sawbones Europe AB, Malmö, Sweden), made of epoxy resin and glass fibre, were used to produce the models. Both the anatomical dimensions and the biomechanical properties of these artificial bones are roughly equivalent to the values for human bone given in the literature [27].

The opening high tibial osteotomy was fixed anteromedially, using an anatomically asymmetrically formed Newclip^®^ Activmotion^®^ osteotomy plate (Haute Goulaine, France) with a length of 61.6 mm (titanium alloy, reference ATGP1-ST) and six locking screws with a diameter of 4.5 mm. All measurements were performed on a servohydraulic testing machine (Instron Prüfsystem 8874H1003, Instron, Darmstadt, Germany) with a biaxial force transducer for axial and torsional loads (up to 10 kN, up to 100 Nm, precision 0.5%). The machine was controlled using the software FTStartUP V.7.22 (Fast Track Start UP) and Instron MAX V.9.2. (Multi axis test controller; INSTRON, Darmstadt, Germany).

**Figure 1:**
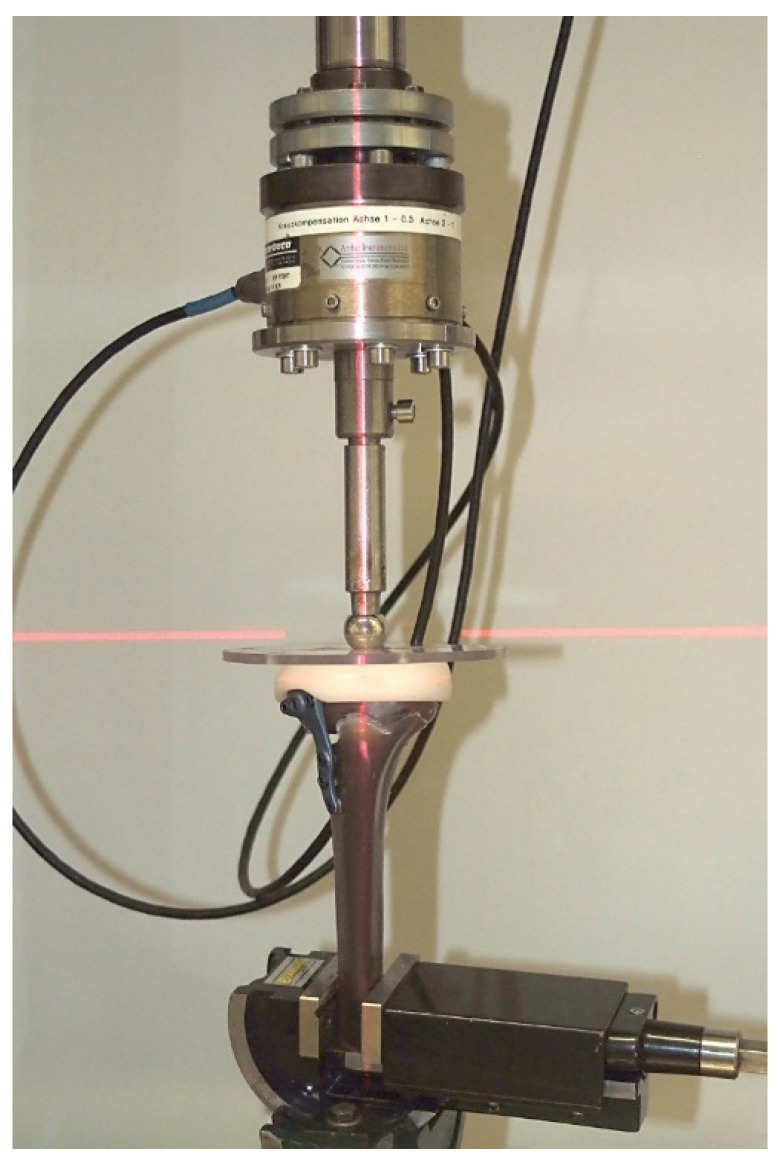
Showing the experimental design of the Instron test system with the mounted test medium with Activmotion osteotomy plate.

**Figure 2:**
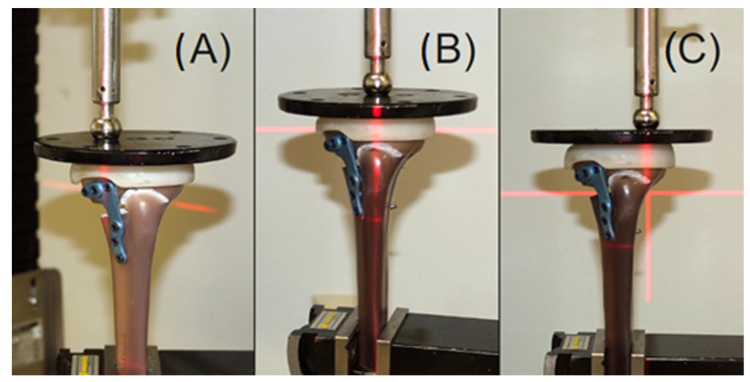
Showing the alignment of the test specimen without a contralateral cortical bone defect in (**A**) regular alignment (RA), (**B**) moderate valgus alignment (VA1), and (**C**) pronounced valgus alignment (VA2).

In order to achieve a physiological force distribution on the tibial plateau of the model, a plastic holder was fitted to its surface. This is connected to a round metal disc in which three cones are incorporated to take up the force transmitter (Figure 1 and Figure 2).

In order to determine the force distribution on the two joint components of the tibial plateau, a round plastic disc of the metal disc fixed to the fixation device for force transmission was reproduced with the corresponding cylindrical drill holes, and 50% of the material weight was distributed on two precision laboratory balances. Then, the force transmitter was placed in each of the three cavities to show the different alignments, and mean values were determined from seven repetitions. Regular alignment produced a percentage force distribution of 63% in the medial compartment, compared with 37% in the lateral compartment. A balanced load distribution of 50% of the load on both joint segments was measured in the case of moderate valgus alignment (VA1). In the case of pronounced valgus alignment (VA2), the load distribution was reversed to a percentage load share of 38% in the medial and 62% in the lateral compartment. The application of force via the medial hole corresponds to a loading in normal, regular leg alignment (RA), over the central hole a moderate valgus alignment (VA1), and over the lateral hole a pronounced valgus alignment (VA2).

**Figure 3:**
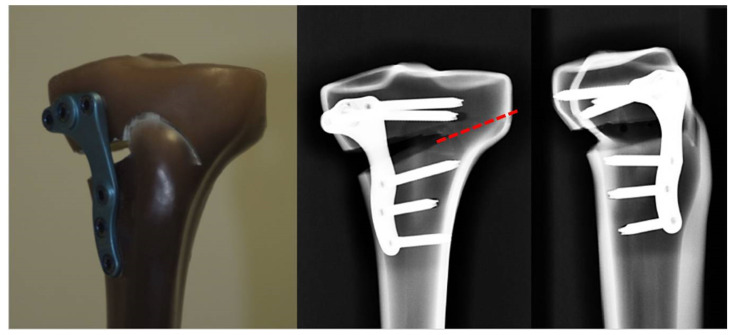
Showing the test specimen with Activmotion osteotomy plate. (**Left**) view from the front; (**centre**) anterior–posterior radiograph; (**right**) mediolateral radiograph. The red dashed line shows the position of the lateral hinge fracture in frontal plane.

Five test models consisting of the above-mentioned materials were prepared in the same way and by the same surgeon. Along previously inserted guide rails made of Kirschner wires, a medial wedge removal [26] with a 10-mm base was performed with an oscillating saw. Within the context of preliminary experiments, it was found that spreading apart the osteotomy gap repeatedly led to failure of the lateral bone bridge, so that excision of a bone wedge with a base of 10 mm was performed instead of spreading, to enable comparability with other studies [14,19]. Then, to produce a clinically established biplanar osteotomy, the tibial tuberosity was sawn around at 130° to the first osteotomy [14,28]. This was followed by the alignment and fixation of the plate with screws in accordance with the manufacturer’s recommendations and checking of the correct preparation in a CT scan (Figure 3). After trials, the lateral hinge fracture was sawed in the course of the pre-existing 130° osteotomy.

**Figure 4:**
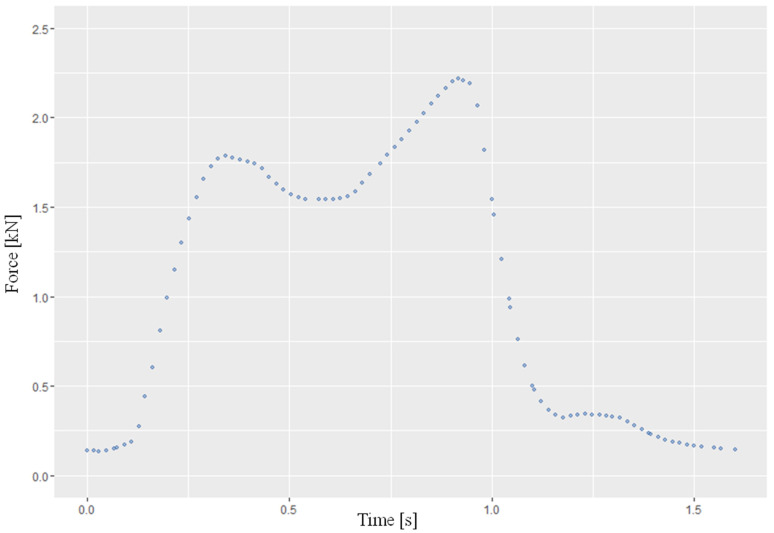
Showing the programmed force profile during one cycle according to Bergmann [29]. (Axes: Force; Time).

A dual-peak force profile during one cycle was programmed (Figure 4), which corresponds to the vertical load during the stance phase when walking [29]. The maximum load that acts in the knee joint during normal walking corresponds to around three times the body weight [30,31,32], so a maximum load of 2.4 kN was applied in this study. A total number of 10,000 cycles with a cycle duration of 1.6 sec was aimed for (a total of 267 min). This number of cycles is based on the average total number of steps of an adult under 65 years of age per day, with an average of 9797 steps [33].

The three runs of the different alignment tests were initially performed (VA1, RA, VA2) with an intact lateral bone bridge of 10 mm and then with a fractured bone bridge (VA1*, RA*, VA2*). All six different test runs followed the same test protocol on five specimens.

**Figure 5:**
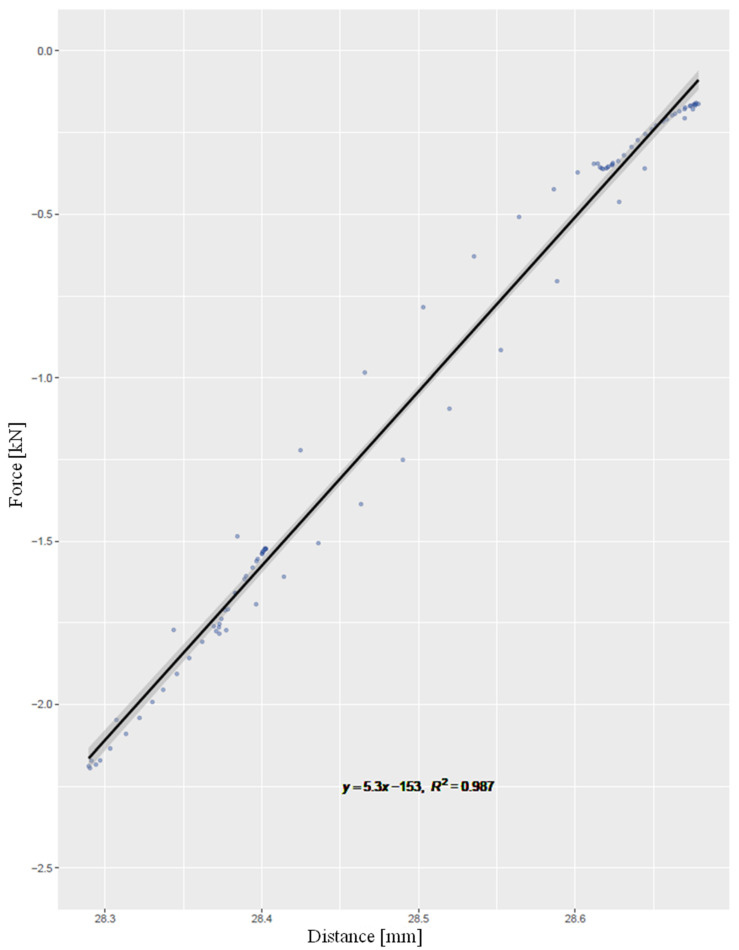
Shows the best-fit line of the force-distance relationship, the gradient of which represents the rigidity (Axes: Force, Distance).

The values are stated as mean ± standard error of the mean (SEM). Initially, graphs were prepared to present the parameters measured over the number of cycles and extreme values of the interfragmentary motion [in mm] and rigidity [in kN/mm] were calculated. The rigidity corresponded to the gradient of the best-fit line of the force–distance function (Figure 5). For statistical analysis with the aid of the program R, the Shapiro–Wilk test and the Wilcoxon rank sum test for independent samples were performed. As it was assumed that the data roughly follow a function of the type y=a(logx)+b, a regression analysis was performed and the coefficient of determination R^2^ was determined. The level of significance was set at 0.05.

## 3. Results

There were no cases of implant loosening or failure. In one specimen, a lateral hinge fracture occurred after 1100 cycles.

**Figure 6:**
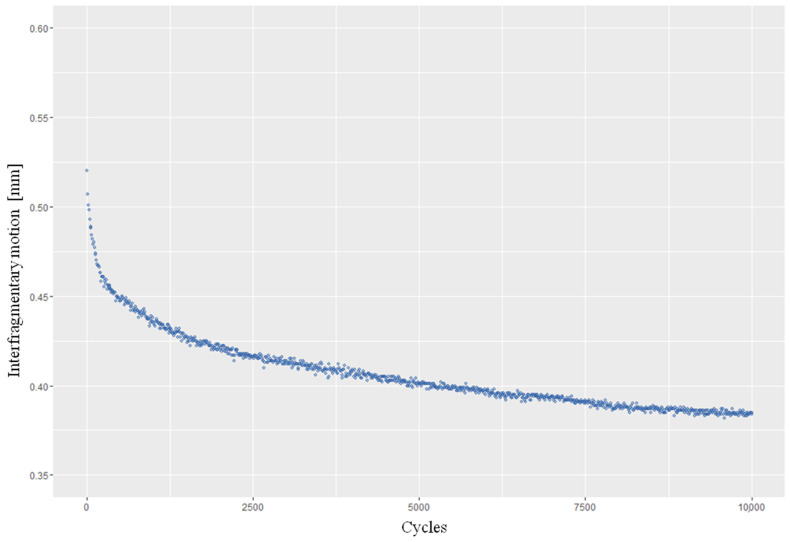
Interfragmentary motion over an exemplary measurement run of 10,000 cycles with 2.4 kN. (Axes: Interfragmentary motion; Cycles).

**Figure 7:**
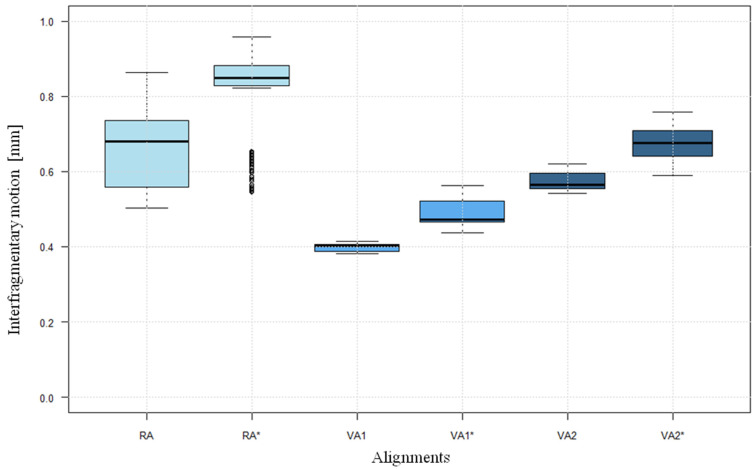
Boxplots of the interfragmentary motion (mean of the cycles 8000 to 10,000 in each case). (Axes: Interfragmentary motion; Alignments), * lateral hinge fracture group.

Interfragmentary motion showed a similar course in all test models (Figure 6). It decreases over 10,000 cycles, with the greatest change at the beginning of the measurement. All the measurements performed tended towards a certain small interfragmentary motion without reaching a complete standstill of the system. Both the level of the means and the maximum values of the motion at different alignments are significantly different (*p* < 0.05). The average interfragmentary motion in RA was 0.668 mm ± 0.098, compared with 0.409 mm ± 0.046 in VA1 and 0.630 mm ± 0.072 in VA2 (Table 1). In the case of a “lateral hinge fracture”, in all alignments the interfragmentary motion was significantly greater than in the case of intact contralateral cortical bone (*p* < 0.05) (Figure 7).

**Figure 8:**
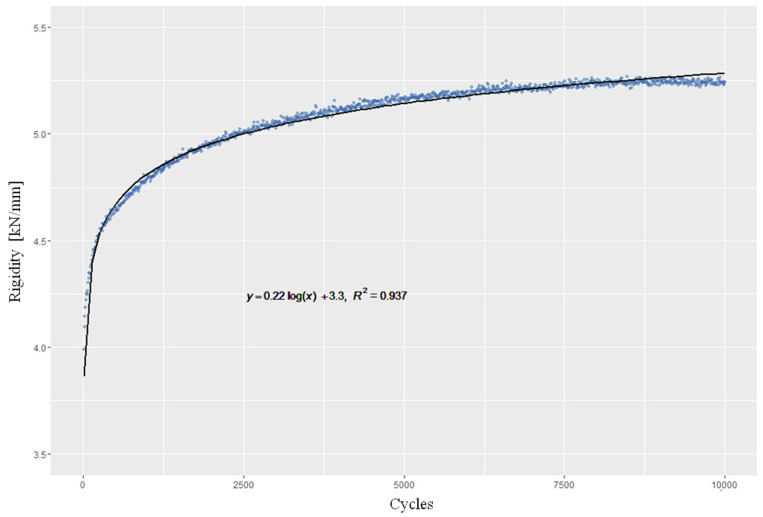
Rigidity over 10,000 cycles with 2.4 kN based on an exemplary measurement. (Axes: Rigidity; Cycles).

**Figure 9:**
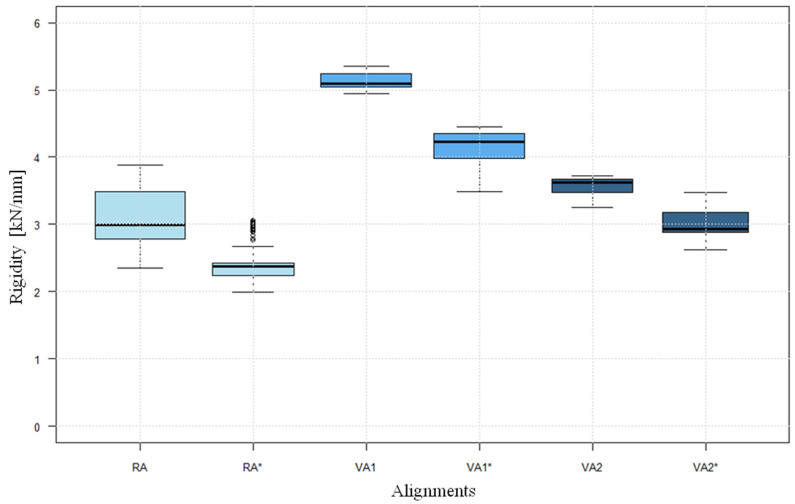
Boxplots of the rigidity (determined as the mean of the cycles 8000 to 10,000). (Axes: Rigidity; Alignments), * lateral hinge fracture group.

The behaviour of rigidity shows a similar course in the different alignments and defect situations. The graphic presentation showed a great similarity to the function y=a(logx)+b (Figure 8). Both the level achieved and the mean value for rigidity are at different levels in the different alignments and defect situations (Table 2). At 4.972 kN/mm ± 0.254, the highest mean rigidity was achieved in VA1, while VA2 at 3.277 kN/mm ± 0.301 and RA at 3.084 kN/mm ± 0.416 showed a lower rigidity. In the presence of a “lateral hinge fracture”, the rigidities achieved in RA* at an average of 2.278 kN/mm ± 0.188 and in VA1* at 3.918 kN/mm ± 0.360 were significantly lower than in the test models with intact cortical bone (*p* < 0.05) (Figure 9).

## 4. Discussion

The main result of the present study is that interfragmentary motion is dependent upon alignment in the frontal plane after HTO and that a lateral hinge fracture leads to an increase in this motion.

The ideal degree of correction after HTO is the subject of considerable debate. A knee aligned in excessive valgus can lead to problems in the patellofemoral joint and to a rapid degeneration of the lateral joint cartilage, while a neutral alignment may lead to progression of deformity, pain and early failure [34]. Alignment in the frontal plane is only part of the important biomechanical factors in planning an HTO. In fact, planning must be three-dimensional and should include posterior tibial tilt of the knee (slope) [35], patella position [36], and medial ligament tension [15], among others.

Some studies have already shown that the cartilage pressure in the affected compartment of the knee joint increases as soon as the mechanical axis is displaced [5,32]. The simplified measurements of the force distribution on the tibial plateau with the aid of two laboratory balances revealed that, even in regular leg alignment, a strictly fifty–fifty force distribution on the two components of the tibial plateau does not occur and thus confirm the results of previous studies [6,7,8,37]. While in Kumar et al. between 66% and 82% of the load is carried on the medial compartment when walking [8], in Zhao et al. it is on average 55% [37]. The present study produced a medial load distribution between these two studies of around 63% in regular alignment (RA). A balanced load distribution was demonstrated in moderate valgus alignment (VA1), and, in the case of pronounced valgus alignment, the force redistribution was even reversed to a loading of 62% in the lateral compartment. In the study of Agneskirchner et al., the force was not applied strictly centrally, but through the Fujisawa point at 62% of the mediolateral transverse diameter of the tibial head [19], which corresponds to the model of moderate valgus alignment in the study presented here.

The smallest interfragmentary movements were observed in moderate valgus alignment (VA1), followed by a pronounced valgus alignment (VA2) and regular alignment (RA). A possible mechanism could be derived biomechanically; the stiffness is the quotient of the change in force to the change in displacement, i.e., in this test the ratio of maximum cyclic force to interfragmentary motion. Since the applied cyclic force was the same for all tests (2.4 kN), different stiffness values result in the deformation path, the interfragmentary motion. As a consequence of indirect proportionality, the stiffness thus increases with decreasing deformation and vice versa. For the tests, the force application points on the force transducer were selected in such a way that the test conditions simulated regular, moderate valgus and severe valgus alignment, which could also be confirmed on the basis of the preliminary tests using two laboratory balances. The balanced force distribution in VA1 on the medial and lateral tibial plateau could thus serve as an explanation for the mechanically most stable alignment with high stiffness and low interfragmentary motion. An interfragmentary motion of up to 0.5 mm is ideal for the formation of a callus with maximum mechanical rigidity in animal experiments according to Claes et al. [18]. Only small interfragmentary movements (approx. 0.2 mm) are necessary for the stimulation of callus formation, and relatively large interfragmentary movements of up to around 1 mm can be tolerated [38].

In the present study, all alignments, both with and without “lateral hinge fracture”, had an average interfragmentary motion below the critical level for reliable bone healing. In all alignments, significantly greater movements occurred in the presence of a “lateral hinge fracture” than with an intact lateral bone bridge, again without exceeding the critical degree of 1 mm interfragmentary motion [38]. In the study conducted by Agneskirchner et al., the most stable implant showed the lowest interfragmentary movements of below 0.1 mm medially and below 0.6 mm laterally, although this was only at a semi-physiological force of 1120 N [19]. In all of the plates investigated in the study published by Kim et al., there was a higher interfragmentary motion of 1.81 mm ± 1.06 to 2.70 mm ± 1.38 at 2000 N axial force loading in a similar experimental design, but using porcine tibiae and a maximum load of 2000 N [21]. In addition, a dissertation with a similar experimental design at a cyclical load of up to a maximum of 1760 N showed that the TomoFix plate had an interfragmentary motion of up to 0.73 mm medially and 0.96 mm laterally [39]. Here, too, there was an increase in interfragmentary motion by 1.22–1.53 mm after lateral hinge fracture [39]. In contrast to this, the plate investigated in the present study also showed an interfragmentary motion sufficient for bone healing in the case of lateral hinge fracture.

In the study conducted by Maas et al., the rigidity of the osteosynthesis showed a slight increase as a result of the compaction of the composite material, as also seen in the present study [25]. The vertical and lateral rigidities were on average 2000 N/mm and 1930 N/mm for the TomoFix plate, compared with 2367 N/mm and 3133 N/mm for the Contour Lock plate, at a considerably higher number of cycles of at least 60,000, but a lower maximum force than in the present study and thus a lower rigidity [25].

The study of Stoffel et al. presents the difference in rigidity of the Puddu and TomoFix plate in the case of intact and fractured lateral contralateral cortical bone of composite tibiae under static pressure. Here, average values of 1349 N/mm and 1701 N/mm were found in the case of intact, compared with 462 N/mm and 910 N/mm in the case of fractured contralateral cortical bone [26], which is considerably lower than in the present study with at least 3084 N/mm in the case of intact and 2278 N/m in the case of defective contralateral cortical bone.

Limitations of the present study include the use of composite tibiae. Their biomechanical properties and anatomical geometry are roughly consistent with those of human bones [27], but are more representative of a younger population [26] and less so for patients with initial osteoarthritis of the knee or even osteoporosis, so that their stability and load-bearing capacity may be overestimated. On the basis of the consistent material properties and the associated small interindividual differences [27], the closely specified surgical technique and the identical surgeon, the osteotomy is highly reproducible and, also due to a precision of the testing machine of 0.5%, influences of material properties or methodology can be considered to be minimal. As a whole, the comparison with other studies shows that there is currently no standardised protocol for the testing of plate systems for corrective osteotomy on the tibia. In all of the available studies, the number of test cycles, the level of loading and the position of force application vary. The method used for measuring interfragmentary motion also differs. This necessarily leads to different results and limits the comparability of the data. Nakamura et al. show that the anteromedial plating without filling the gap with the TomoFix plate can lead to more pendular micromotion than a lateral positioning with bone-substitute insertion [40]. This experimental study was performed only on the effect of axial force in the fully extended position of the knee, which never produces the pendular micromotion. The shift from femur to tibia as during walking, stair climbing or sitting down is not considered. Takeuchi et al. performed mechanical testing of the TomoFix plate at knee flexion angles of 0° and 10° and concluded that a medial plate position is biomechanically superior to an anteromedial position [41]. In conclusion the selection of plate, filling the gap and plate position should be made critically.

This is an in vitro study, so influences, such as muscle traction or ligament tension, were not taken into account [26]. In addition, only the force application of normal walking was considered. Higher force impacts that may possibly occur when standing up or stumbling over [7] were not investigated. The one-day simulation without pendular micromotion cannot guarantee long-term durability without stress fractures of the plate or screws due to pendular micromotion. Moreover, the determination of total rigidity was performed.

## 5. Conclusions

In the case of the anteromedially positioned fixed-angle plate investigated here, leg alignment had a significant influence on interfragmentary motion and rigidity. In all alignment situations, a lateral hinge fracture led to an increase in interfragmentary motion as well as to a decrease in the rigidity measured. However, both values were below the accepted limits for sufficient bone healing. Despite its small size and the anteromedial positioning, the implant investigated here appears to be forgiving with regard to this complication.

## Figures and Tables

**Table 1:** Showing the interfragmentary motion in [mm] of the cycles 1–10,000.

	Min	Max	MW	Median	SD
RA	0.503	1.718	0.668	0.683	0.098
RA*	0.545	2.114	0.862	0.871	0.089
VA1	0.382	2.138	0.416	0.409	0.046
VA1*	0.437	1.891	0.518	0.510	0.060
VA2	0.475	2.517	0.630	0.616	0.072
VA2*	0.588	2.586	0.716	0.710	0.080

RA = regular alignment, VA1 = moderate valgus alignment, VA2 = pronounced valgus alignment, * = with lateral hinge fracture, Min = minimum, Max = maximum, MW = mean, SD = standard deviation.

**Table 2:** Shows the rigidity of the cycles 1–10,000 in [kN/mm].

	Min	Max	MW	Median	SD
RA	1.226	3.879	3.084	2.985	0.416
RA*	1.005	3.057	2.278	2.287	0.188
VA1	0.945	5.353	4.972	5.000	0.254
VA1*	1.135	4.452	3.918	4.016	0.360
VA2	0.866	3.723	3.277	3.324	0.301
VA2*	0.864	3.711	2.864	2.860	0.288

RA = regular alignment, VA1 = moderate valgus alignment, VA2 = pronounced valgus alignment, *= with lateral hinge fracture, Min = minimum, Max = maximum, MW = mean, SD = standard deviation.

## Data Availability

The data presented in this study are available on request from the corresponding author.
